# A Circular Letter to the Physicians of Kentucky on Medical Reform

**Published:** 1842-04-23

**Authors:** 


*A Circular Letter to the Physicians of Kentucky.* 12mo. pp. 12.

A number of the physicians of Kentucky recently met at Frankfort in that state, to discuss the important subject of a reform in the mode of conferring medical degrees. At this conference, the following resolution was unanimously adopted:

"*Resolved*, That the interests of the medical profession, and of the public in general, would be promoted by the establishment of a board of examining physicians, who shall meet annually for the purpose of conferring diplomas on all candidates who may be found worthy, upon a rigorous examination."

A circular address has been issued, in support of the foregoing resolution, in which the whole subject is very ably discussed. We hail with pleasure this movement, and believe that it will effect important changes in our system of medical education. The principle contended for we have repeatedly advocated in this journal, and we shall heartily co-operate with the efforts of our brethren of the profession throughout the country to give it practical force. We have always thought that much would be gained by separating the power to confer degrees from the business of teaching; and we have little doubt that such a separation will in time be effected. The practical consequences of a change of system, will perhaps not in all respects meet the anticipations of our friends in Kentucky. It may be doubted whether "the immediate results of the establishment of a board of state examiners would be to throw medical teaching, in a great measure, into the hands of the practitioners throughout the state." We believe, on the contrary, that it will continue to be centralized in the large cities, where the facilities for study are greater, although probably, as is suggested in the circular, the amount of teachers will be multiplied, and the classes be advantageously diminished in number. The circular judiciously disclaims feelings of hostility towards existing schools. These may usefully co-exist with an examining board, as in Ireland, where there are a dozen medical schools and but one college of physicians. The objects aimed at, are simply a higher and uniform degree of qualification for a diploma, and the privilege of candidates to qualify themselves with the teachers or lecturers of their own selection. We are sincerely glad that the physicians of Kentucky are awakening the attention of the profession to this subject.

